# Survey of equine veterinarians regarding primary equine back pain in the United States

**DOI:** 10.3389/fvets.2023.1224605

**Published:** 2023-07-26

**Authors:** Marianne E. Marshall-Gibson, Matthew G. Durham, Kathryn A. Seabaugh, Valerie J. Moorman, Dora J. Ferris

**Affiliations:** ^1^Front Range Equine Performance LLC, Berthoud, CO, United States; ^2^Independent Researcher, Marina, CA, United States; ^3^Department of Clinical Sciences, College of Veterinary Medicine and Biomedical Sciences, Colorado State University, Fort Collins, CO, United States; ^4^Orthopaedic Research Center, Colorado State University, Fort Collins, CO, United States; ^5^Department of Large Animal Medicine Surgery and Lameness Service, Veterinary Teaching Hospital University of Georgia, Athens, GA, United States; ^6^Summit Equine Inc., Gervais, OR, United States

**Keywords:** back-pain, rehabilitation, survey, sports medicine, equine, impinging spinous processes, kissing spine

## Abstract

Back pain is a common complaint, clinical finding and performance limiting factor in sport horses. This study sought to gather current veterinary trends in the diagnosis, treatment and management of primary equine back pain in the United States. A 22 question survey was distributed electronically to equine practitioners through AAEP and ACVSMR listservs and through closed social media groups. The survey was open from April 20, 2022 to July 5, 2022. Responses were analyzed using Microsoft excel pivot tables. Ninety-seven survey responses were obtained and analyzed. Respondents reported the clinical signs most frequently relayed to them by the owner/rider/trainer of horses diagnosed with primary back pain were behavioral issues and poor performance. Most common diagnostic tests reported were radiography of the spinous processes, thoraco-lumbar vertebral bodies, and transcutaneous ultrasound of the thoraco-lumbar region. Most common pathologies reported were impinging dorsal spinous processes, degenerative sacro-iliac joint disease, and osteoarthritis in lumbar or thoracic articular process joints. In regards to impinging spinous process (“kissing spine”) treatments, 72.2% of respondents recommended surgery only after non-surgical treatments failed, and 14.6% of respondents never recommended surgery. The majority (82%) of respondents reported some level of improvement in clinical signs of primary back pain with rehabilitation alone. To date, there has been no consensus or discussion about common abnormalities, diagnostic tests, treatments or management options for primary equine back pain in the United States. Results of this survey are a starting point showing current trends in diagnosis, treatment and management of primary equine back pain among equine practitioners in the United States showing 82% of practitioners using rehabilitation as a component of treatment.

## 1. Introduction

Back pain is a common complaint and clinical finding in poorly performing equine athletes, with an estimate of up to 94% of ridden horses experiencing back pain (1). It is typically characterized as either primary (directly related to insults or pathology in the thoraco-lumbo-sacral regions) or secondary (related to compensation for non-primary back injuries or pathologies) back pain (2–4). The etiology of primary back pain can be quite complex, originating from bone, joint, ligament, tendon and muscle injury, or a combination of these (3–5). With increasing availability and affordability of mobile imaging equipment, along with growing continuing education training, equine practitioners have been more readily able to diagnose primary causes of equine back pain. A more accurate diagnosis has led to advances in treatment options as well as the widespread incorporation of rehabilitation into the management of affected horses (3–7).

The most reported and commonly diagnosed etiology of primary back pain in horses is impinging spinous processes (ISP) or “kissing spine.” The treatment and management of horses with ISP can range from conservative management to more invasive surgical techniques. While the studies describing the surgical treatment of ISPs show favorable results with between 72 and 91% of horses undergoing surgery returning to some level of performance (8–14), there is a surprising lack of literature, systematic reviews or randomized clinical trials, evaluating efficacy of alternative treatment and management options for ISP.

Despite being a common topic discussed among performance horse veterinarians, there is still a general lack of randomized clinical studies and general consensus on best approaches to diagnose, treat, and manage primary equine back pain. This lack of evidence could lead veterinarians toward treatments and management bias based on practitioner preference and possibly geographical location (2).

The study sought to gather current veterinary trends in the diagnosis, treatment and management of primary equine back pain and ISPs in the United States.

## 2. Materials and methods

A survey consisting of 22 questions (see Supplementary material) was distributed electronically using Google Forms^1^ to equine practitioners through the American Association of Equine Practitioners (AAEP) and American College of Veterinary Sports Medicine and Rehabilitation (ACVSMR) listservs and through closed veterinary social media groups: Equine Vet-2-Vet, Equine Lameness Vets, and Women in Equine Practice. Due to the American College of Veterinary Surgeons (ACVS) diplomate contact policy, mass distribution of the survey to this demographic was not able to be performed. Based on the number of equine veterinarians in each group, it is estimated that approximately 3,500 equine veterinarians received access to the questionnaire. The survey was open from April 20, 2022 to July 5, 2022. Responses were analyzed using Microsoft Excel pivot tables. Only fully completed questionnaires were included in the results and each response required a unique identifier to eliminate duplicate responses.

## 3. Results

### 3.1. Demographics

A total of 97 complete survey responses were obtained and analyzed. The regions of the United States represented are shown in Table 1. Primary practice or general practitioners comprised 58% of the respondents while 30% practiced at specialty/s opinion practices or University Teaching Hospitals. Ninety-two percent of respondents represented practices with a predominantly equine (>76% of patients) caseload. The top three breeds reported to be seen by respondents were Warmbloods (90%), Thoroughbreds (88%), and Quarter Horses (84%). Other breeds had minimal representation (<10% each). The disciplines of horses serviced by the respondents based on percentage is shown in Table 2. This table shows a wide variety of disciplines being served by respondents to the current survey, with a slightly higher representation of hunter/jumper and dressage horses.

**Table 1:** Regions of the United States where practitioners predominantly practice.

Region of United States respondents predominantly practice	# Respondents
Midwest (OH, IN, IL, WI, MI, MO, IA, MN, KS, NE, SD, ND)	13
Mountain West (MT, WY, ID, CO, UT, NV)	20
Northeast (PA, NY, NJ, CT, MA, RI, VE, NH, ME, DC, DE, MD)	13
Pacific Coast (WA, OR, CA)	11
Southeast (WV, VA, NC, SC, KY, TN, AR, LA, MS, AL, GA, FL)	31
Southwest (OK, TX, NM, AZ)	9
Total	97

**Table 2:** Percentage of disciplines represented in respondents’ practices.

Discipline	0	<10%	10–25%	26–50%	51–75%	76–99%	100%	Total
AQHA and similar competitions	14	50	21	6	5	1	0	97
Cutting/reining	23	54	16	1	3	0	0	97
Rodeo (team/tie-down/calf roping)	38	36	16	5	2	0	0	97
Working ranch	40	38	14	2	2	1	0	97
Barrel racing	18	40	29	6	3	1	0	97
Eventing	9	37	35	9	4	3	0	97
Endurance	52	40	4	1	0	0	0	97
Hunter/jumper	6	16	26	29	14	5	1	97
Dressage	5	24	39	21	6	2	0	97
Racetrack	60	23	7	3	2	2	0	97
Pleasure/trail	8	29	37	17	5	1	0	97
Polo	62	32	3	0	0	0	0	97
Driving	71	23	1	1	1	0	0	97
Other	78	13	5	0	1	0	0	97

### 3.2. Clinical signs and diagnosis of primary back pain

Respondents were asked to characterize the signs of primary back pain in their practice. Forty-eight respondents reported between 10 and 25% of patients showing signs of primary back pain, twenty-four respondents reported low (<10%) numbers of patients, eleven respondents reported 26–50% of patients having primary back pain, and fourteen respondents reporting >50% of patients having primary back pain in their practice.

**Figure 1:**
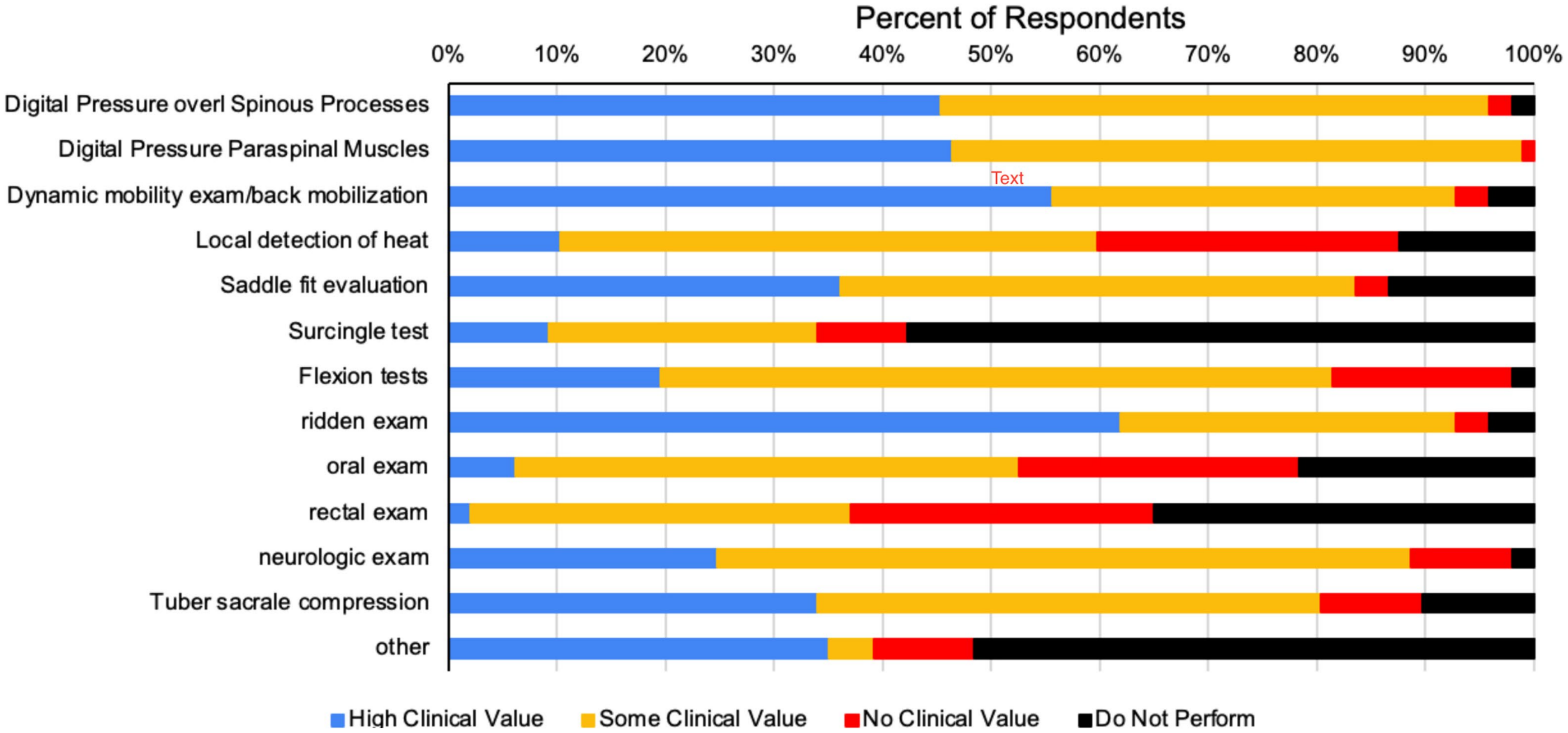
Perceived clinical value of various clinical tests performed by respondents to diagnose primary equine back pain.

Clinical signs respondents recalled being reported to them by the owner, rider or trainer of horses diagnosed with primary back pain are summarized in Table 3. The most frequently reported clinical signs recalled being relayed to respondents by the owner/rider/trainer of horses diagnosed with primary back pain were behavioral issues and poor performance. Additional responses included sore to back palpation, unwillingness to stand still, or bucking/rearing. Respondents were then asked to evaluate various clinical tests for their perceived value when used to diagnose primary equine back pain. Respondents reported digital pressure over dorsal spinous processes and paraspinal muscles, dynamic mobility/back mobilization exam and ridden exam to have the highest clinical values when diagnosing primary equine back pain (Figure 1).

**Table 3:** Frequency of clinical signs reported to respondents by the owner, rider or trainer of horses diagnosed with primary back pain.

Complaint	Always (100%)	Frequently (51–99%)	Somewhat frequently (26–50%)	Infrequently (<25%)	Never	Total
Bruxism	0	1	16	56	24	97
Tail swishing/altered tail carriage	2	29	39	22	5	97
Aggressive behavior	0	6	28	50	13	97
Bunny hopping gait behind	1	25	42	22	7	97
Difficult transitions	3	35	40	17	2	97
Focal heat	0	1	9	55	32	97
Hindlimb lameness	3	28	33	28	5	97
Forelimb lameness	0	5	20	55	17	97
Difficulty sliding/stopping	2	11	17	38	29	97
Change in jumping style	3	22	32	32	8	97
Kicking out	3	23	40	28	3	97
Unwilling to go forward under saddle	5	30	41	17	4	97
Difficulty when saddling	4	28	46	17	2	97
Difficulty holding canter leads	2	30	43	19	3	97
Difficulty bending	5	21	48	20	3	97
Loss of topline/muscle atrophy	4	23	41	28	1	97
Refusing jumps	1	17	34	38	7	97
Loss of impulsion	5	39	33	18	2	97
Missing lead changes	2	26	43	23	3	97
Girthy/cinchy	2	36	47	12	0	97
Other	1	3	6	14	73	97

**Figure 2:**
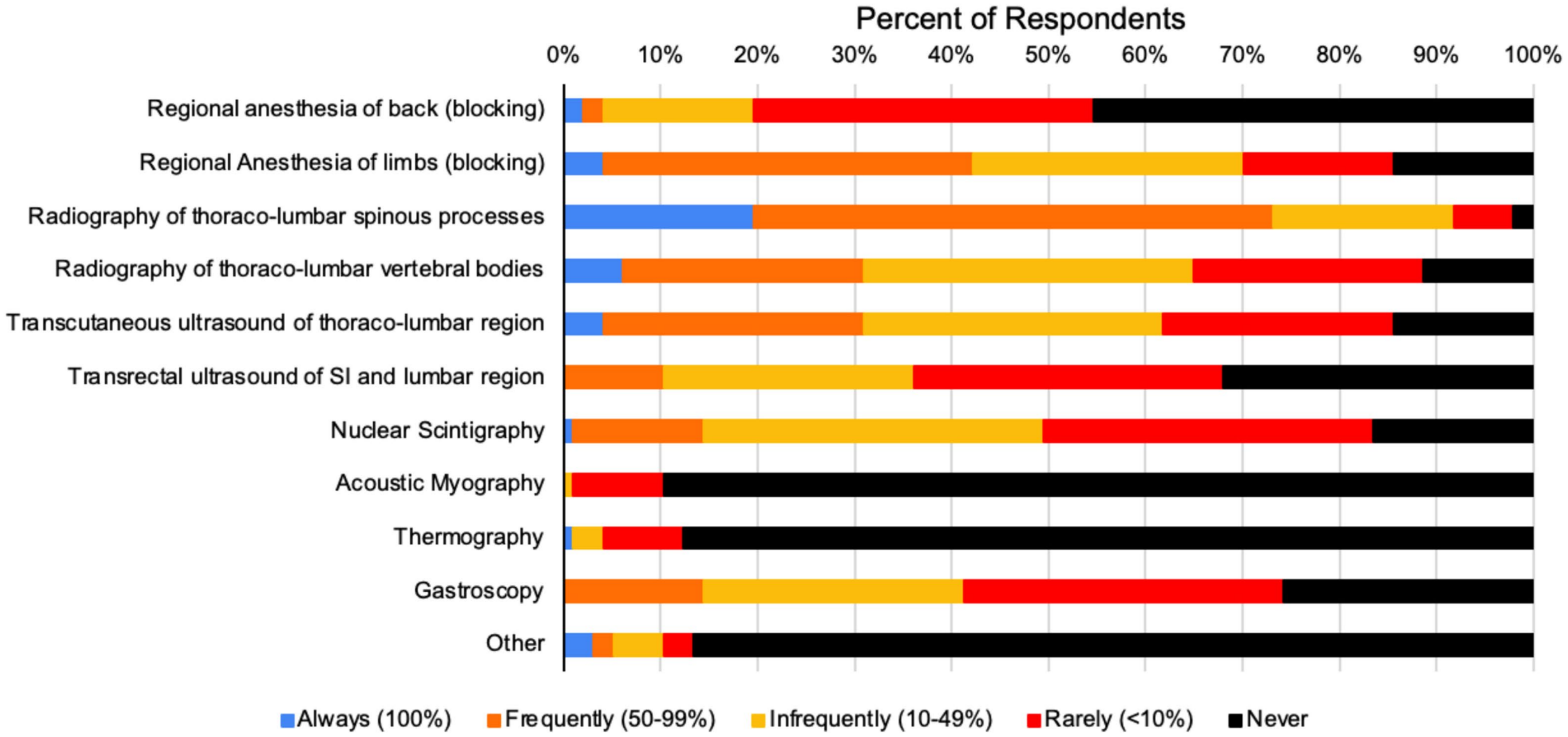
Frequency with which respondents use various modalities to diagnose primary equine back pain.

**Figure 3:**
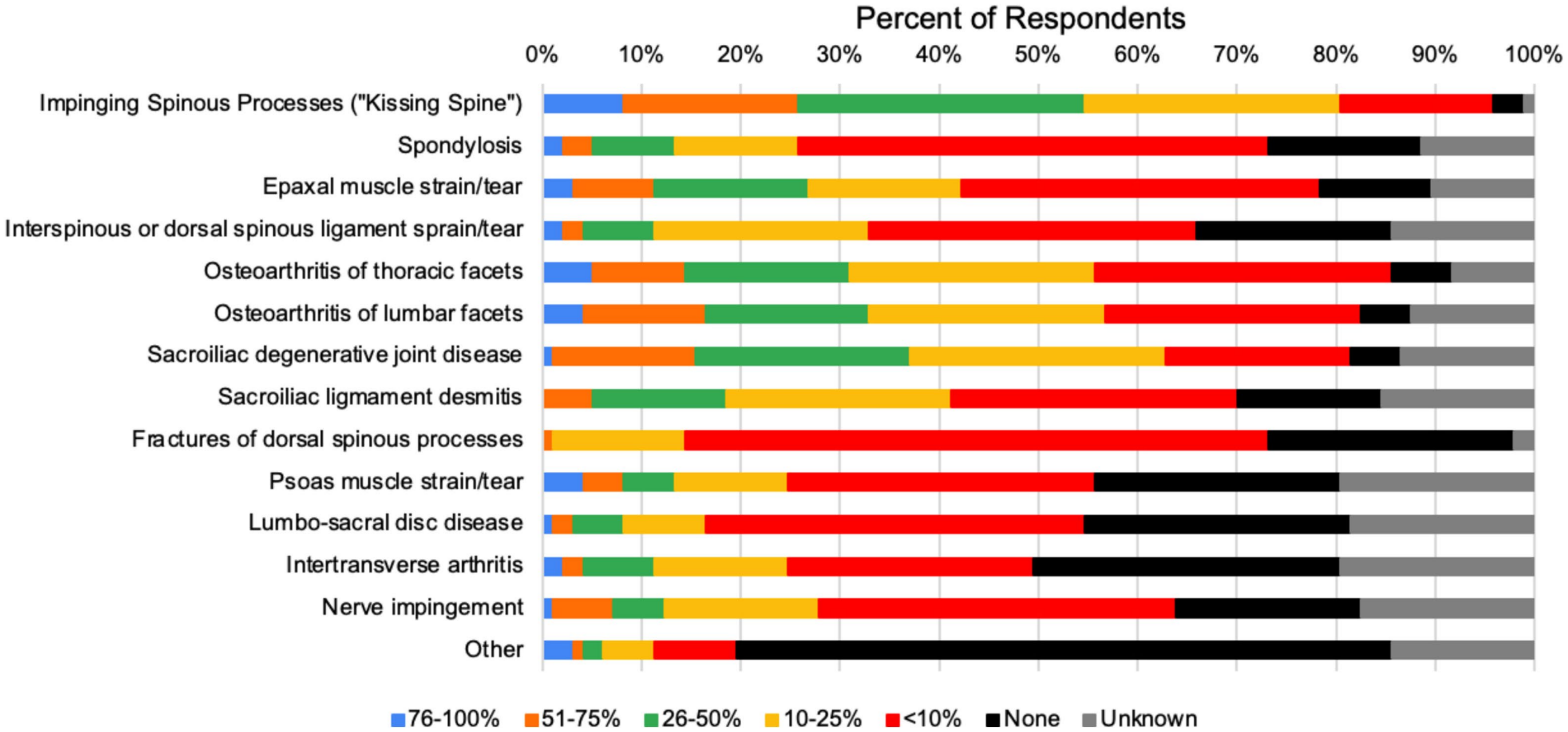
Pathologies reported with relative frequency in horses with primary equine back pain.

When asked what diagnostic tests respondents used to diagnose primary back pain, the most frequently utilized diagnostic tests were radiography of the spinous processes, thoraco-lumbar vertebral bodies, and transcutaneous ultrasound of the thoraco-lumbar region (Figure 2). Pathologies reported to be seen in patients associated with primary equine back pain are shown in Figure 3. Of the pathologies represented in the survey, 78 respondents reported impinging spinous processes in >10% of patients, 61 respondents reported degenerative sacro-iliac joint disease in >10% of patients, and 55 and 54 respondents reported osteoarthritis in lumbar or thoracic articular process joints in >10% of patients, respectively.

### 3.3. Treatment and management of primary equine back pain

**Figure 4:**
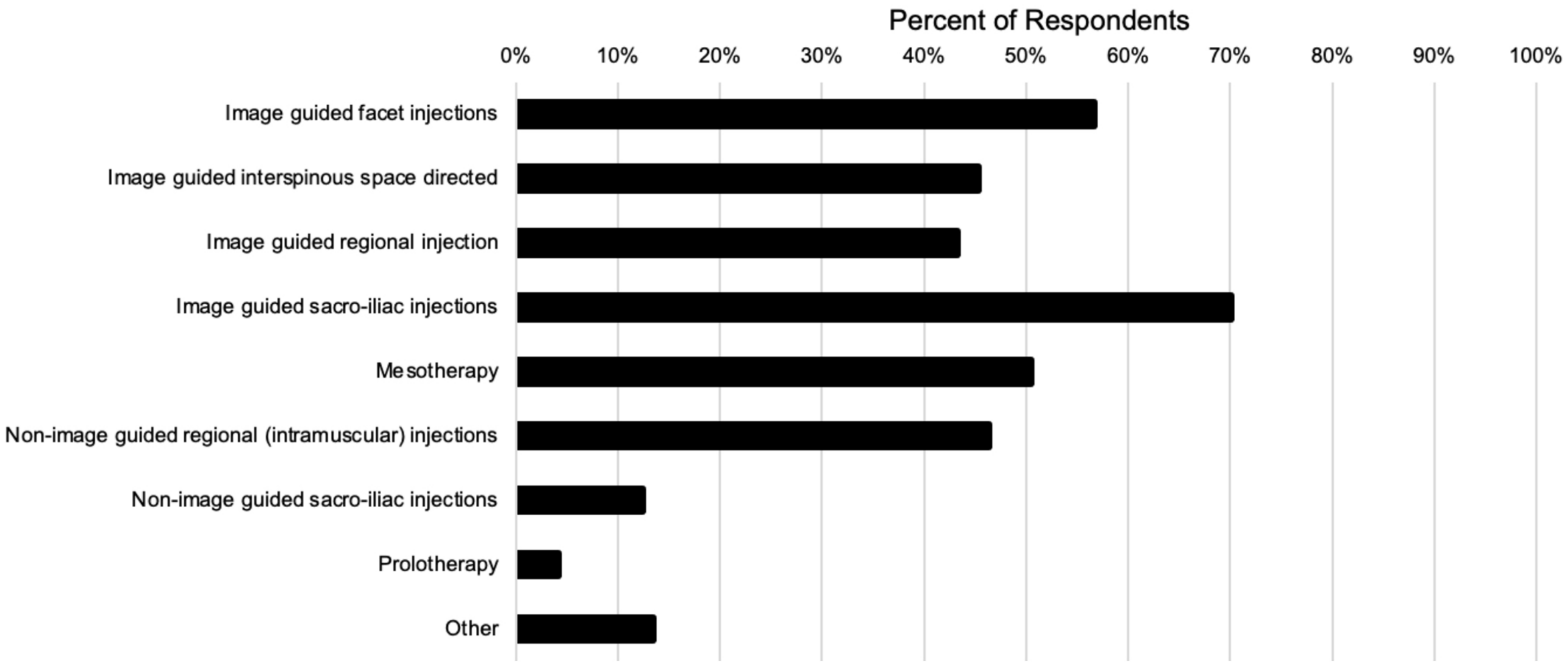
Injection techniques used by respondents for treatment of primary equine back pain.

The survey asked questions pertaining to the treatment modalities used to manage primary equine back pain. Table 4 shows a summary of the therapies recommended for first line treatment of primary back pain. The most frequently recommended first line therapies included Rehabilitation (44% always recommended), Shockwave (46% frequently recommend), NSAIDs or Chiropractic (45% frequently recommend), and Acupuncture (44% frequently recommend). When asked about the efficacy of various treatment modalities in treating primary equine back pain, respondents reported rehabilitation (64%), shockwave (49%), and local intra-articular injections (43%) to be either always (100%) effective or effective 50–99% of the time (Table 5). The techniques commonly used for injection treatment of primary back pain are summarized in Figure 4. With regards to treating the Sacro-iliac region, image guided injections had a higher reported use (70%) versus non-image guided (12%) injections. Regional injections had similar response rates of 43 and 46% for image guided and non-image guided, respectively.

**Table 4:** Therapies recommended by respondents for first line treatment of primary back pain with relative frequency.

Treatment	Always (100%)	Frequently (50–99%)	Infrequently (10–49%)	Rarely (<10%)	Never	Total
Local intramuscular injections	3	28	29	21	16	97
Local intra-articular injections	2	37	26	18	14	97
Mesotherapy	3	18	33	15	28	97
Prolotherapy	0	3	5	9	80	97
Shockwave therapy	10	45	18	11	13	97
Bisphosphonates	2	16	25	22	32	97
Non-steroidal anti-inflammatory drugs (NSAIDs) - oral or topical	12	44	22	13	6	97
Surgery (in the case of ISP/“kissing spine”)	2	12	19	40	24	97
Gabapentin	1	9	21	33	33	97
Methocarbamol	7	35	28	20	7	97
Chiropractic	12	44	23	11	7	97
Acupuncture	10	43	28	11	5	97
Laser therapy	4	23	15	30	25	97
Pulsed electro-magnetic field (PEMF)	0	13	20	21	43	97
Functional electrical simulation (FES)	2	4	7	21	63	97
Rehabilitation	43	26	17	9	2	97
Other	6	2	4	1	84	97

**Table 5:** Assessment of efficacy of various treatment modalities for primary equine back pain with relative effectiveness.

Treatment modality	Always effective (100%)	Effective (50–99%)	Somewhat effective (10–49%)	Ineffective (<10%)	Do not perform/recommend	Total
Local intramuscular injections	3	32	30	9	23	97
Local intra-articular injections	3	39	30	5	20	97
Mesotherapy	2	22	32	9	32	97
Prolotherapy	1	3	7	2	84	97
Shockwave therapy	6	42	25	8	16	97
Bisphosphonates	2	13	36	13	33	97
Non-steroidal anti-inflammatory drugs (NSAIDs) - oral or topical	2	25	51	14	5	97
Surgery (in the case of ISP/“kissing spine”)	1	31	31	8	26	97
Gabapentin	0	11	23	22	41	97
Methocarbamol	1	23	41	22	10	97
Chiropractic	3	37	37	9	11	97
Acupuncture	4	36	41	7	9	97
Laser therapy	4	18	25	16	34	97
Pulsed electro-magnetic field (PEMF)	1	10	24	15	47	97
Functional electrical stimulation (FES)	2	6	11	7	71	97
Rehabilitation	20	42	24	7	4	97
Other	4	4	2	3	84	97

**Figure 5:**
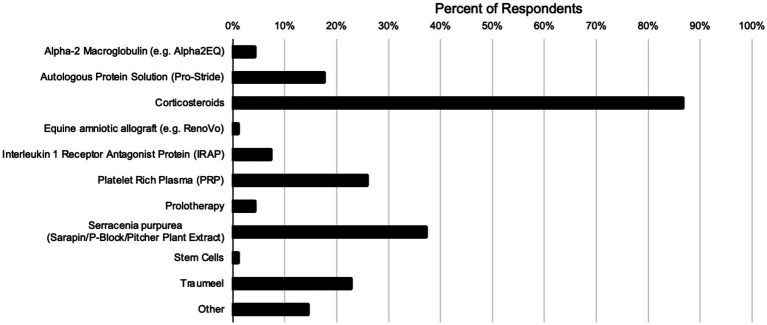
Substances used by respondents in primary back pain injection treatments.

Respondents were asked which substances they used for local injection treatments of primary back pain (Figure 5). The most frequently reported was corticosteroids (84 responses) followed by *Sarracenia purpurea* (36 responses). Other responses included lidocaine/local anesthetic (3 responses), Adequan (2 responses), and vitamin B (2 responses).

**Figure 6:**
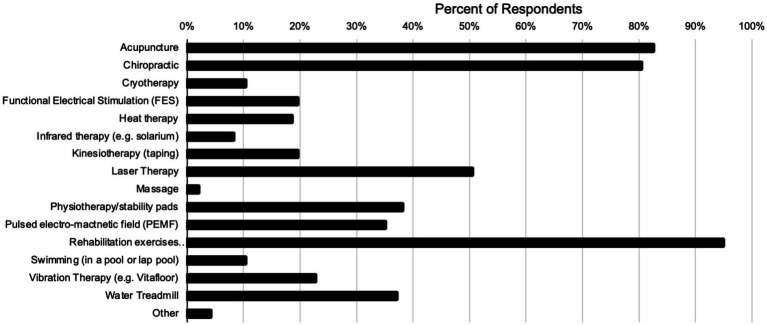
Recommended rehabilitation and management modalities by respondents for primary equine back pain.

Additionally, respondents were asked which modalities they recommend for rehabilitation of patients with primary back pain (Figure 6). The most frequently recommended therapies by respondents were Rehabilitation exercises (95%), Acupuncture (82%), Chiropractic (80%), and laser therapy (51%).

### 3.4. Treatment and management of impinging spinous processes

**Figure 7:**
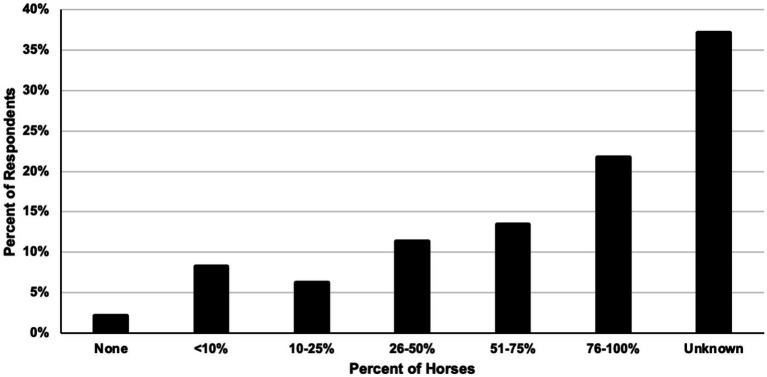
Percent of horses that require follow up non-surgical treatments after impinging spinous process surgery in respondents’ practices.

In regards to impinging spinous process (“kissing spine”) treatments, 70% of respondents recommended surgery only after non-surgical treatments failed, and 14.6% of respondents never recommended surgery. When surgery was recommended, the majority of respondents (64.6%) left the type of surgical procedure performed for treatment of impinging spinous processes up to the surgeon, while some (16.7%) preferred interspinous ligament desmotomy. Of respondents that had followed horses after ISP surgery (59%), 34% said less than 50% of horses show improvement in presenting clinical signs immediately after surgery. Additionally, 22% of respondents felt 76–100% of horses that had undergone ISP surgery required follow up non-surgical treatments, while only 2% felt horses did not need follow up intervention (Figure 7).

**Figure 8:**
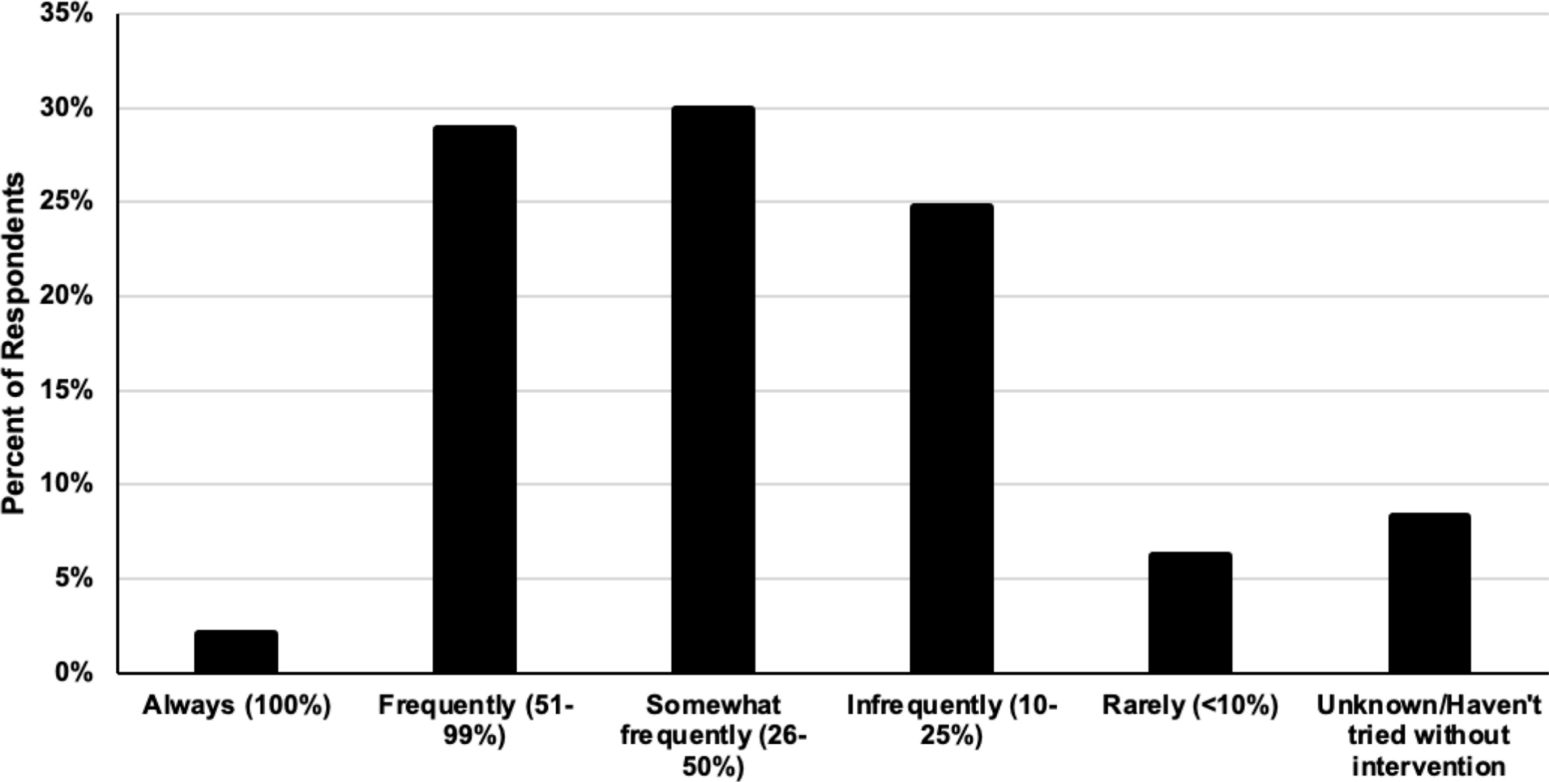
Relative frequency respondents see horses that show improvement in clinical signs related to impinging spinous processes with rehabilitation alone (i.e., without medical or surgical intervention).

The respondents were asked two questions regarding rehabilitation in the management of horses with ISP. In regards to timing of rehabilitation when horses were undergoing surgery, 49% of respondents said they recommended rehabilitation before surgery, 67% said they recommended rehabilitation after surgery, and 54% they recommended rehabilitation before and after surgery (respondents were able to choose more than one option for this question). Furthermore, the majority (82%) of respondents reported some level of improvement in clinical signs of primary back pain with rehabilitation alone (Figure 8).

## 4. Discussion

The diagnosis, treatment and management of equine back pain has evolved over the past decade with advancements in diagnostic imaging techniques, treatment and management options, and more focused incorporation of rehabilitation. The current survey is the first attempt known by the authors to gather information on current veterinary trends regarding primary equine back pain in the United States.

A limited number of responses were gathered; therefore, a true generalization of trends cannot be made. However, the geographic distribution of respondents is similar to the relative population of equine veterinarians across the United States, indicating a good sampling of current trends (15). A limitation of the survey was respondents represented a variety of equine veterinarians with different experience levels and practice types. In 2011 Haussler et al. reported back pain and associated problems were seen in anywhere from 0.9 to 94% of ridden horses (1). The large variance was expected to be due to veterinarian experience in recognizing and diagnosing primary back pain as well as the breeds being examined as previously demonstrated (5). Likewise, respondents of this survey were not asked about the percentage of performance horse work conducted in their respective practices, nor about their experience or advanced training in diagnosing and treating primary back pain. In addition, breeds represented in the study were mostly limited to Warmbloods, Thoroughbreds and Quarter Horses, hence a generalization to the entirety of the horse population in the United States cannot be made.

When considering clinical signs respondents reported owners riders and trainers associating with primary back pain, they frequently reported non-specific signs of poor performance and behavioral issues similar to previous studies (2, 5). Respondents leaned more towards subjective measures of back palpation and ridden exam to have a high clinical value when diagnosing back pain, while a lower percentage relied on more objective measures like regional anesthesia, which was less popular with veterinarians in the United States than previously reported by European veterinarians (2). Similar to previous findings, most frequently used diagnostic modalities for examination of primary back pain were radiography and ultrasonography, where acoustic myography and thermography were almost never employed (2). This is likely due to the expense of equipment and lack of validation of the latter modalities in their ability to consistently diagnose back pain.

Consistent with radiography and ultrasonography being the most frequently utilized diagnostic modalities for primary equine back pain, pathologies most frequently associated with primary equine back pain in this survey were those that could be diagnosed with these diagnostic tools: impinging spinous processes, degenerative sacro-iliac joint disease, and osteoarthritis in lumbar and thoracic articular process joints. This is not surprising given the relative availability, versatility, and portability of this type of equipment in the ambulatory setting. Furthermore, in recent years there has been a large increase in training available to equine practitioners utilizing ultrasound and radiology in the diagnosis of axial skeletal issues.

When considering first line treatments for primary equine back pain, respondents recommended non-invasive treatments (shockwave, chiropractic, acupuncture, NSAIDs) and rehabilitation. This is comparable to findings in the human literature which show high success rates in managing chronic back pain with NSAIDs, physical therapy and chiropractic care (7). In contrast to a survey by Wilson in 2018 which reported mesotherapy to be commonly utilized by 87% of their respondents to manage neck and back pain, only 21% of respondents to this survey reported they used this modality >50% of the time. This may be due to the narrow focus of this study to primary back pain vs. inclusion of neck issues. The same reasoning could explain the difference in respondents reporting the use of certain biologic therapies in that study versus this study where very few veterinarians reported the use of IRAP or Stem Cells in the treatment of primary back pain but were more likely to use PRP and similar products. Consistent with the previous survey of European veterinarians (2), corticosteroids were the most frequently reported to be utilized for injectable treatments of primary equine back pain by veterinarians in the United States with 87% of respondents reporting use in this study and 80% of respondents reporting use in the previous study. This is likely due to the relative cost, widespread availability and perceived effectiveness in treatment and management of back pain versus other biologic therapies that have not been effectively studied in this application.

Looking at modalities employed to manage equine neck and back pain, the most common modalities reported to be effective in this study were less invasive modalities of chiropractic, acupuncture, shockwave, and rehabilitation (6). This finding was in line with Wilson’s 2018 study asking practitioners which modalities they recommended for equine neck and back pain (6). These modalities frequently fall under the jurisdiction of veterinary medicine in the United States, with some variability in state laws allowing lay people to perform these treatments. Additionally, advanced training in acupuncture, chiropractic and rehabilitation is readily available to veterinarians in the United States through structured certification programs offered by a number of institutions. Therefore, veterinarians in the United States are typically familiar with these modalities and their applications due to an increasing number of veterinarians having certifications in one or more of them.

Following trends of other studies (2, 8–14), impinging spinous processes was the most frequently reported pathology associated with primary equine back pain in this survey. This may be due to the increased availability and portability of digital radiography in the field and general comfort of veterinarians with identifying osteopathic lesions compared to soft tissue abnormalities in the back. Although the literature demonstrates high success rates of horses responding to surgical treatment for ISPs (8–14), 72.2% of respondents to the current survey expressed a reluctance to recommend surgery as a first line treatment, with a portion (14.4%) opting to never recommend surgery for ISPs. This response may be due to a range of factors including the lack of experience, access to a surgical center and bias. The reluctance or refusal to recommend surgery may account for the large number of respondents reporting the number of horses requiring follow up non-surgical treatments after ISP surgery being unknown. However, respondents to this survey reported the percentage of horses requiring follow up intervention post-surgery due to suspected recurrent back pain as much larger than previously reported (8). Further conclusions could not be drawn as non-surgical interventions were not specifically defined in the survey to differentiate medical treatments vs. rehabilitation modalities, and the definition was left open to interpretation by respondents (Figure 7; Supplementary material). With regards to the effectiveness of rehabilitation in horses with ISPs in the absence of medical or surgical intervention, the respondents reported some proportion of horses not requiring additional therapies to manage their back pain. To the authors’ knowledge, this is the first report on the perceived effectiveness of rehabilitation in the absence of medical or surgical intervention in the treatment and management of ISPs by veterinarians.

In conclusion, results of this survey are a starting point showing current trends in diagnosis, treatment and management of primary equine back pain among equine practitioners in the United States. Additional investigations directly comparing the efficacy of the various treatment and rehabilitation modalities used to manage primary equine back pain is warranted given the relative frequency with which certain modalities are utilized or recommended by equine veterinarians. Furthermore, there is a precedent for evaluating the long-term effectiveness of rehabilitation in the absence of surgical or medical intervention in the management of horses presenting with clinical ISPs.

## Data availability statement

The raw data supporting the conclusions of this article will be made available by the authors, without undue reservation.

## Author contributions

MM-G conceived and designed the study and was responsible for data collection. MD, KS, VM, and DF contributed to preparing the survey. MM-G and DF contributed to interpretation of data. MM-G drafted the manuscript and all authors edited and revised it critically for content.

## Footnotes

^1^https://www.google.com/forms/about/
