# Supplementary material for: Survey of equine veterinarians regarding primary equine back pain in the United States
Source: Front Vet Sci. 2023 Jul 26;10:1224605. doi: 10.3389/fvets.2023.1224605 (PMC10411723; doi:10.3389/fvets.2023.1224605)
Supplement: Supplementary file 1 [file Data_Sheet_1.pdf]

Supplementary Material: The following pages contain the unaltered questionnaire that was presented to veterinarians to obtain responses that were analyzed and reported for the Survey of Equine Veterinarians Regarding Primary Equine Back Pain in the U.S.A. manuscript.

Survey of primary equine back pain in the U.S.A.

Hello!

This is a survey for equine veterinary practitioners within the United States of America and its Territories. The survey consists of 22 questions and should take about 15 minutes to complete. The goal is to determine 1) the preference of practitioners when treating primary back pain and 2) the preference of practitioners for treating impinging spinous processes ("kissing spine") in horses in the United States. For the purposes of this study "primary back pain" referrs to pain localized to the T3-S5 region ("withers to tail"). Results will be published after analysis.

Note about the survey: Questions 5 and 7-12 require an entry in every row, including the "Other" row. If you do not have an "other" response, please leave the subsequent question space blank.

Thank you for participating and contributing to our knowledge of equine primary back pain!

mmarshall@frequine.com Switch account

Not shared

* Indicates required question

In what region of the U.S. do you predominently practice? *

- Northeast (PA, NY, NJ, CT, MA, RI, VE, NH, ME, DC, DE, MD)
- Southeast (WV, VA, NC, SC, KY, TN, AR, LA, MS, AL, GA, FL)
- Midwest (OH, IN, IL, WI, MI, MO, IA, MN, KS, NE, SD, ND)
- Southwest (OK, TX, NM, AZ)
- Mountain West (MT, WY, ID, CO, UT, NV)
- Pacific Coast (WA, OR, CA)
- Non-contiguous (AK, HI, US Territories)

How would you describe your veterinary practice? *

- General/Primary ambulatory practice
- Specialty/Second opinion referral practice (non-primary)
- Private Referral Hospital
- University Teaching Hospital
- Other:

What percentage of your practice is Equine? *

- <25%
- 26-50%
- 51-75%
- 76-100%

Please rank the top 3 horse breeds that predominate the equine patients in your practice:

	1	2	3
Thoroughbred	○	○	○
Quarter Horse	○	○	○
Warmblood	○	○	○
Standardbred	○	○	○
Saddlebred	○	○	○
Morgan	○	○	○
Tennessee Walker	○	○	○
Draft	○	○	○
Pony	○	○	○
Other	○	○	○

If you have an "other" response to the question above, please specify below. If you have more than one "other" please specify ranking (1-3) as in the question above:

Your answer

What percentage of your patients perform in the following disciplines? *

	0	<10%	10-25%	26-50%	51-75%	76-99%	100%
AQHA and similar competitions	○	○	○	○	○	○	○
Cutting/Reining	○	○	○	○	○	○	○
Rodeo (Team/Tie-down/Calf Roping)	○	○	○	○	○	○	○
Working Ranch	○	○	○	○	○	○	○
Barrel Racing	○	○	○	○	○	○	○
Eventing	○	○	○	○	○	○	○
Endurance	○	○	○	○	○	○	○
Hunter/Jumper	○	○	○	○	○	○	○
Dressage	○	○	○	○	○	○	○
Race Track	○	○	○	○	○	○	○
Pleasure/Trail	○	○	○	○	○	○	○
Polo	○	○	○	○	○	○	○
Driving	○	○	○	○	○	○	○
Other	○	○	○	○	○	○	○

If you have an "other" response to the question above, please specify below. If you have more than one "other" response, please specify with relative percentage of each as in the question above:

Your answer

In your opinion, what percentage of horses have primary back pain at your practice? *

- None
- <10%
- 10-25%
- 26-50%
- 51-75%
- 76-100%

Which of the following clinical signs are reported by the owner/rider/trainer of horses you diagnose with primary back pain? *

	Never	Infrequently (<25%)	Somewhat Frequently (26-50%)	Frequently (51-99%)	Always (100%)
Agressive behavior	○	○	○	○	○
Buruxism	○	○	○	○	○
Tail Swishing/altered tail carriage	○	○	○	○	○
Difficulty when saddling	○	○	○	○	○
Kicking out	○	○	○	○	○
Girthiness/cinchy	○	○	○	○	○
Focal heat	○	○	○	○	○
Loss of topline/muscle atrophy	○	○	○	○	○
Loss of impulsion	○	○	○	○	○
Bunny-hoping gait behind	○	○	○	○	○
Difficult transitions	○	○	○	○	○
Hindlimb lameness	○	○	○	○	○
Forelimb lameness	○	○	○	○	○
Difficulty					

Difficulty sliding/stopping	○	○	○	○	○
Change in jumping style	○	○	○	○	○
Unwilling to forward under saddle	○	○	○	○	○
Difficulty holding canter leads	○	○	○	○	○
Missing lead changes	○	○	○	○	○
Difficulty bending	○	○	○	○	○
Refusing jumps	○	○	○	○	○
Other	○	○	○	○	○

If you have an "other" response to the question above, please specify below. If you have more than one "other" response, please specify with relative frequency of each as in the question above:

Your answer

In your opinion, which of the following clinical test(s) do you find have clinical value in diagnosing primary back pain at your practice? *

	High Clinical Value	Some Clinical Value	No Clinical Value	Do Not Perform
Digital pressure over dorsal spinous processes	○	○	○	○
Digital pressure paraspinal muscles	○	○	○	○
Dynamic mobility exam/back mobilization	○	○	○	○
Local detection of heat	○	○	○	○
Saddle fit evaluation	○	○	○	○
Surcingle test	○	○	○	○
Flexion tests	○	○	○	○
Ridden Exam	○	○	○	○
Oral Exam	○	○	○	○
Rectal Exam	○	○	○	○
Neurologic Exam	○	○	○	○
Tuber sacrale compression	○	○	○	○
Other	○	○	○	○

If you have an "other" response to the question above, please specify below. If you have more than one "other" response, please specify with relative clinical value of each as in the question above:

Your answer

How frequently do you use the following to diagnose primary back pain? *

	Never	Rarely (<10%)	Infrequently (10-49%)	Frequently (50-99%)	Always (100%)
Regional Anesthesia of back (blocking)	○	○	○	○	○
Regional Anesthesia of limbs (blocking)	○	○	○	○	○
Radiography of thoraco-lumbar dorsal spinous processes	○	○	○	○	○
Radiography of thoraco-lumbar vertebral bodies	○	○	○	○	○
Transcutaneous ultrasound of thoraco-lumbar region	○	○	○	○	○
Transrectal ultrasound of Sacro-iliac adn lumbar region	○	○	○	○	○
Nuclear Scintigraphy	○	○	○	○	○
Acoustic Myography	○	○	○	○	○
Thermography	○	○	○	○	○
Gastroscopy	○	○	○	○	○
Other	○	○	○	○	○

If you have an "other" response to the question above, please specify below. If you have more than one "other" response, please specify relative frequency of each as in the question above:

Your answer

In your opinion, what percentage of horses with primary back pain do you find the following pathologies at your practice? *

	None	<10%	10-25%	26-50%	51-75%	76-100%	Unknown
Impinging Spinous Processes ("Kissing Spine")	◯	◯	◯	◯	◯	◯	◯
Spondylosis	◯	◯	◯	◯	◯	◯	◯
Epaxal muscle strain/tear	◯	◯	◯	◯	◯	◯	◯
Interspinous or dorsal spinous ligament sprain/tear	◯	◯	◯	◯	◯	◯	◯
Osteoarthritis of thoracic facets	◯	◯	◯	◯	◯	◯	◯
Osteoarthritis of lumbar facets	◯	◯	◯	◯	◯	◯	◯
Sacroiliac degenerative joint disease	◯	◯	◯	◯	◯	◯	◯
Sacroiliac ligmament desmitis	◯	◯	◯	◯	◯	◯	◯
Fractures of dorsal spinous processes	◯	◯	◯	◯	◯	◯	◯
Psoas muscle strain/tear	◯	◯	◯	◯	◯	◯	◯
Lumbo-sacral disc disease							

Intertransverse arthritis	○	○	○	○	○	○	○
Nerve impingement	○	○	○	○	○	○	○
Other	○	○	○	○	○	○	○
Other	○	○	○	○	○	○	○

If you have an "other" response to the question above, please specify below. If you have more than one "other" response, please specify relative percentage of each as in the question above:

Your answer

Which of the following therapeutics do you recommend for first line treatment of primary back pain? *

	Never	Rarely (<10%)	Infrequently (10-49%)	Frequently (50-99%%)	Always (100%)
Local intramuscular injections	○	○	○	○	○
Local intra-articular injections	○	○	○	○	○
Mesotherapy	○	○	○	○	○
Prolotherapy	○	○	○	○	○
Shockwave therapy	○	○	○	○	○
Bisphosphonates (e.g. Clodronate/OsPhos, Tiludronate/Tildren, Zoledronate)	○	○	○	○	○
Non-steroidal anti-inflammatory drugs (NSAIDs) - oral or topical	○	○	○	○	○
Surgery (in the case of impinging spinous processes or "kissing spine")	○	○	○	○	○
Gabapentin	○	○	○	○	○
Methocarbamol	○	○	○	○	○
Chiropractic	○	○	○	○	○
Acupuncture	○	○	○	○	○

Laser therapy	○	○	○	○	○
Pulsed electro-magnetic field (PEMF)	○	○	○	○	○
Functional Electrical Simulation (FES)	○	○	○	○	○
Rehabilitation	○	○	○	○	○
Other	○	○	○	○	○

If you have an "other" response to the question above, please specify below. If you have more than one "other" response, please specify relative frequency of each as in the question above:

Your answer

In your opinion, which of the following treatment modalities are effetive in treating primary back pain at your practice? *

	Do not perform/recommend	Ineffective (<10%)	Somewhat effective (10-49%)	Effective (50-99%)	Always effective (100%)
Local intramuscular injections	○	○	○	○	○
Local intra-articular injections	○	○	○	○	○
Mesotherapy	○	○	○	○	○
Prolotherapy	○	○	○	○	○
Shockwave therapy	○	○	○	○	○
Bisphosphonates (e.g. Clodronate/OsPhos, Tiludronate/Tildren, Zoledronate)	○	○	○	○	○
Non-steroidal anti-inflammatory drugs (NSAIDs) - oral or topical	○	○	○	○	○
Surgery (in the case of impinging spinous processes or "kissing spine")	○	○	○	○	○
Gabapentin	○	○	○	○	○
Methocarbamol	○	○	○	○	○
Chiropractic	○	○	○	○	○
Acupuncture	○	○	○	○	○

Laser therapy	○	○	○	○	○
Pulsed elctro-magnetic field (PEMF) therapy	○	○	○	○	○
Functional electrical etimmulation (FES)	○	○	○	○	○
Rehabilitation	○	○	○	○	○
Other	○	○	○	○	○

If you have an "other" response to the question above, please specify below. If you have more than one "other" response, please specify relative effectiveness of each as in the question above:

Your answer

Which of the following techniques do you use for injection treatment of primary back pain? *

- [ ] Non-image guided regional (intramuscular) injections
- [ ] Image guided regional injection
- [ ] Image guided interspinous space directed
- [ ] Image guided facet injections
- [ ] Image guided sacro-iliac injections
- [ ] Non-image guided sacro-iliac injections
- [ ] Mesotherapy
- [ ] Prolotherapy
- [ ] Other:

What substances are you using for injection treatments of primary back pain? *

- [ ] Corticosteroids
- [ ] Interleukin 1 Receptor Antagonist Protein (IRAP)
- [ ] Platelet Rich Plasma (PRP)
- [ ] Autologous Protein Solution (Pro-Stride)
- [ ] Alpha-2 Macroglobulin (e.g. Alpha2EQ)
- [ ] Equine amniotic allograft (e.g. RenoVo)
- [ ] Amniotic acellular reconstitutable liquid implant (e.g. EquusCell AniMatrX D™)
- [ ] Stem Cells
- [ ] Serracenia purpurea (Sarapin/P-Block/Pitcher Plant Extract)
- [ ] Traumeel
- [ ] Prolotherapy
- [ ] Other:

Which of the following do you recommend in rehabilitaiton and management of primary back pain? *

- [ ] Acupuncture
- [ ] Chiropractic
- [ ] Laser Therapy
- [ ] Kinesiotherapy (taping)
- [ ] Functional Electrical Stimulation (FES)
- [ ] Pulsed electro-mactnetic field (PEMF)
- [ ] Physiotherapy/stability pads
- [ ] Rehabilitation exercises (core strengthening dynamic mobility exercises)
- [ ] Water Treadmill
- [ ] Swimming (in a pool or lap pool)
- [ ] Cryotherapy
- [ ] Heat therapy
- [ ] Vibration Therapy (e.g. Vitafloor)
- [ ] Infrared therapy (e.g. solarium)
- [ ] Other:

Page 1 of 2

Next

Clear form

Never submit passwords through Google Forms.

This form was created inside of Front Range Equine Performance. Report Abuse

Google Forms

Survey of primary equine back pain in the U.S.A.

mmarshall@frequine.com Switch account

Not shared

* Indicates required question

Impinging Spinous Processes Treatment & Management

The last 7 questions pertain to aim 2) the preference of practitioners for treating impinging spinous processes ("kissing spine") in horses in the United States.

At what timepoint in treatment of impinging spinous processes ("kissing spine") do you recommend surgery? *

- Never
- First line treatment
- After regional anesthesia of back (blocking)
- After nuclear scintigrapy (bone scan)
- When non-surgical treatments fail
- Other:

In your opinion, what percentage of horses show improvement in presenting clinical signs after non-surgical treatment at your practice? *

- None
- <10%
- 10-25%
- 26-50%
- 51-75%
- 76-100%

When recommending surgery for impinging spinous processes ("kissing spine"), which procedure do you recommend? *

- Interspinous ligament desmotomy (ISLD or "lig-snip")
- Subtotal ("cranial wedge) ostectomy - part of affected impnging spinous process is removed to create a "shark fin" appearance
- Total ostectomy - complete removal of affected impinging spinous process
- I refer to a surgical facility and the surgeon chooses
- Other:

In your opinion, what percentage of horses show improvement in presenting clinical signs immediately after surgery (i.e. within 2 weeks) for impinging spinous processes ("kissing spine") at your practice? *

- None
- <10%
- 10-25%
- 26-50%
- 51-75%
- 76-100%
- Unknown

In your opinion, what percentage of horses that have undergone surgery for impinging spinous processes ("kissing spine") require follow up non-surgical treatments at your practice? *

- None
- <10%
- 10-25%
- 26-50%
- 51-75%
- 76-100%
- Unknown

When recommending surgery for impinging spinous processes ("kissing spine"), do you recommend rehabilitation? *

	Never	Rarely (1<10%)	Infrequently (10-25%)	Somewhat frequently (26-50%)	Frequently (51-99%)	Always (100%)
Before surgery	○	○	○	○	○	○
After surgery	○	○	○	○	○	○
Before & after surgery	○	○	○	○	○	○

In your opinion, do horses diagnosed with impinging spinous processes ("kissing spine") show improvement in clinical signs with rehabilitation alone (i.e. without medical or surgical intervention) at your practice? *

- ○ Never
- ○ Rarely (<10%)
- ○ Infrequently (10-25%)
- ○ Somewhat frequently (26-50%)
- ○ Frequently (51-99%)
- ○ Always (100%)
- ○ Unknown/Haven't tried without intervention

Page 2 of 2

Back Submit Clear form

Never submit passwords through Google Forms.

This form was created inside of Front Range Equine Performance. Report Abuse
